# The Influence of Insulin Resistance on Outcomes in Hospitalizations for Alcohol-Related Liver Disease: A Nationwide Study

**DOI:** 10.7759/cureus.42964

**Published:** 2023-08-04

**Authors:** Victory Okpujie, Opeyemi Tobalesi, Fidelis Uwumiro, Amaka C Ugoh, Elsie O Osiogo, Olawale Abesin, Oluwatobi A Olaomi, Chimaobi O Nwevo, Tosin Ayantoyinbo, Franklin Ejeagha

**Affiliations:** 1 Internal Medicine, Central Hospital Benin, Benin, NGA; 2 Internal Medicine, College of Health Sciences, University of Ilorin, Ilorin, NGA; 3 Internal Medicine, Our Lady of Apostles Hospital, Jos, NGA; 4 Internal Medicine, University of Benin Teaching Hospital, Benin, NGA; 5 Internal Medicine, Ahmadu Bello University Teaching Hospital, Zaria, NGA; 6 Internal Medicine, Royal Cornwall Hospital NHS Trust, Truro, GBR; 7 Radiology, University of Ibadan, Ibadan, NER; 8 Medicine and Surgery, University of Calabar Teaching Hospital, Calabar, NGA; 9 Internal Medicine, Obafemi Awolowo College of Health Sciences, Olabisi Onabanjo University, Ife, NGA; 10 Internal Medicine, University of Nigeria Teaching Hospital, Enugu, NGA

**Keywords:** chronic liver disease, inpatient mortality, acute liver failure (alf), alcohol-related liver disease, insulin resistance

## Abstract

Background

Alcoholic liver disease (ALD) is known to contribute to the onset of insulin resistance (IR), which has been speculated to worsen the outcome of the disease. This study examines the impact of IR on the severity and outcomes of hospitalizations for ALD.

Methods

A retrospective study was performed using the combined 2016 to 2018 Nationwide Inpatient Sample. All admissions for ALD were included. The association between IR and the clinical and resource utilization of hospitalizations for ALD was analyzed using multivariate regression models to adjust for confounding variables.

Results

About 294,864 hospitalizations for ALD were analyzed. Of these, 383 cases (0.13%) included a secondary diagnosis of IR, while the remaining 294,481 hospitalizations (99.87%) were considered as controls. The incidence of IR in the study was 1.3 per 1000 admissions for ALD. IR was not associated with any significant difference in the likelihood of mortality (adjusted odds ratio (aOR): 1.10, 95% confidence interval (CI): 0.370-3.251, p=0.867), acute liver failure, or the incidence of complications (aOR: 0.83, 95% CI: 0.535-1.274, p<0.001). However, the study found that ALD hospitalizations with IR had longer hospital stays (7.3 days vs. 6.0 days: IRR, 1.17; 95% CI, 1.09-1.26; p<0.001) and higher mean hospital costs ($91,124 vs. $65,290: IRR, 1.32; 95% CI, 1.20-1.46; p<0.001) compared to patients without IR.

Conclusion

IR alone does not worsen the outcomes of patients with ALD, and its association with longer hospital stays and higher mean hospital costs could be due to other confounding factors.

## Introduction

Alcoholic liver disease (ALD) is a pressing global health concern that has a significant impact on healthcare systems worldwide, resulting in substantial annual costs amounting to billions of dollars [1,2]. The association between insulin resistance (IR) and liver cirrhosis was first observed by Bohan and later named hepatogenous diabetes by Megyesi et al. [3,4]. Recent research has validated this association, with alcoholic cirrhotic men exhibiting a significantly higher risk of developing type 2 diabetes compared to non-cirrhotic individuals [5].

Chronic alcohol exposure impairs insulin and insulin-like growth factor signaling in the liver, leading to severe hepatic IR. This inhibition of insulin signaling is further augmented by the activation of phosphatases, such as phosphatase and tensin homologue (PTEN), resulting in a failure of cells to transmit signals downstream through Erk/mitogen-activated protein kinase (MAPK) and phosphatidylinositol 3-kinase (PI3K), which are essential for DNA synthesis, liver regeneration, growth, survival, cell motility, glucose utilization, plasticity, and energy metabolism. Moreover, the production of lipids, such as ceramides, during steatohepatitis leads to increased IR, oxidative stress, and injury [6-8]. The correlation between ALD and IR is a significant cause of concern, as it potentially exacerbates the already substantial health risks associated with ALD. Therefore, it is essential to conduct extensive research on the underlying mechanisms and clinical relevance of IR in ALD, to enable better management of this complex and challenging condition. The increasing prevalence of ALD worldwide, coupled with its high mortality rate, further emphasizes the need for attention to this comorbidity [9]. Understanding the impact of IR on clinical outcomes in ALD can lead to the development of targeted interventions that can help slow down the progression of the disease, improve patient care experience, or mitigate risks. Unfortunately, few studies of high quality examine the prevalence of IR in ALD or its effects on clinical outcomes and disease progression.

The primary objective of this research is to assess the prevalence of IR among individuals diagnosed with ALD and investigate its potential impact on clinical outcomes. This study aims to address the existing knowledge gaps by contributing national-level data on IR prevalence within the context of ALD and exploring potential associations between IR and various clinical outcomes. Specifically, the study intends to investigate whether the presence of IR is linked to poorer outcomes for ALD patients, such as elevated mortality rates, prolonged hospitalization durations, increased total hospital charges (THC), and a higher incidence of inpatient complications.

We hypothesize that IR will exhibit an association with unfavorable clinical outcomes in patients diagnosed with ALD. By conducting this investigation, we seek to pave the way for further research in this area and potentially shed light on the role of IR as a prognostic indicator in the context of ALD. Through a comprehensive analysis and the generation of evidence-based findings, this study aspires to contribute valuable insights that can inform clinical decision-making and facilitate the development of targeted interventions aimed at improving patient outcomes in ALD cases complicated by IR.

## Materials and methods

Design and data source

The study's data were extracted from the Nationwide Inpatient Sample (NIS) databases, covering the period between 2016 and 2018. The NIS database, overseen by the Agency for Healthcare Research and Quality, stands as the largest publicly accessible repository in the United States, encompassing comprehensive information on various hospitalizations. Its design aims for representativeness, encompassing all nonfederal acute-care hospitals in the nation, achieved through a stratified sampling approach based on diverse hospital characteristics. These characteristics include hospital size, teaching status, ownership, geographic location, and urban or rural status, wherein a 20% sample of hospitals is selected within each stratum. All hospitalizations from the chosen hospitals are meticulously recorded and weighted to ensure national representativeness of the data.

The NIS database comprises a comprehensive array of patient demographics, diagnoses (including one primary diagnosis and up to 25 secondary diagnoses), and procedures (encompassing up to 15 primary and secondary procedures). It incorporates important hospital characteristics, such as ownership, bed size, teaching status, urban/rural location, and regional distribution. In addition, the database includes vital information on healthcare resource utilization, including length of stay (LOS), total hospitalization charges (THC), and patient discharge disposition. Notably, the NIS database encompasses information on all hospital stays, encompassing even Medicare advantage patients, who represent a substantial portion, up to 30%, of the entire Medicare beneficiary population. In this context, the principal diagnosis signifies the primary reason for hospitalization from which the primary study cohort (ALD) is defined, while secondary diagnoses encompass any additional conditions (including IR), inclusive of complications occurring during the index hospitalization. The structural and content description of the NIS database demonstrates notable similarities to those detailed in a previously published paper, attesting to its reliability and rendering it a robust and credible data source for the present study [10].

Inclusion criteria and study variables

**Figure 1:**
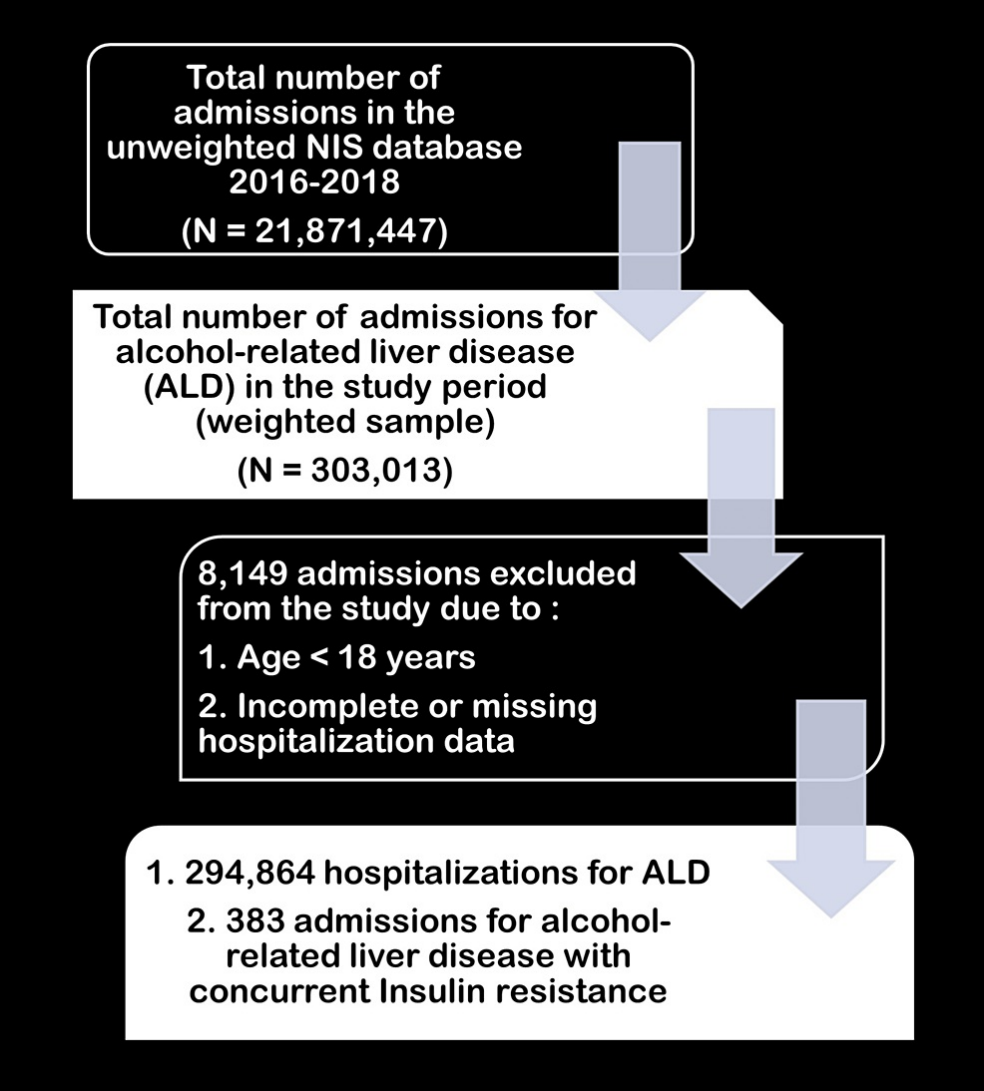
Study selection criteria NIS: Nationwide Inpatient Sample; ALD: alcoholic liver disease

The study population encompassed all adult hospitalizations for alcohol-related liver disease (ARLD) recorded in the combined NIS database spanning the years 2016 to 2018. These hospitalizations were classified into two groups based on the presence or absence of IR, as indicated by the International Statistical Classification of Diseases and Related Health Problems 10th Revision (ICD-10) code E34.322. Hospitalizations for minors and those with incomplete ore missing data were excluded from the study (Figure 1). The NIS database comprises pre-defined variables, including mortality, which was captured as a dichotomous variable labeled "DIED," and variables denoting THC and LOS, both of which serve as numerical indicators reflecting hospital costs and duration of hospitalization, respectively.

To identify potential confounding variables and inpatient complications, we utilized specific codes from the ICD-10-CM/PCS codes. These codes were utilized to ascertain the presence of various conditions, including variceal bleeding, hepatic encephalopathy, hepatorenal syndrome, systemic inflammatory response syndrome (SIRS) or sepsis, liver cirrhosis, liver cancer, malnutrition, and admissions to critical care units.

Outcome measures

The primary outcome was mortality among hospitalizations for ALD complicated by IR compared to ALD alone. Secondary outcomes were acute liver failure (ALF) (ICD-10-CM K70.4 code), odds of inpatient complications, mean LOS, and total hospital costs. We also studied baseline prevalence rate and sociodemographic characteristics for ALD hospitalizations with and without IR.

Statistical analysis

The statistical analysis was carried out using Stata version 16.0-MP (StataCorp LLC, College Station, TX USA). We analyzed the sociodemographic characteristics of the study cohort dichotomized by IR and estimated the prevalence of IR in ALD hospitalizations included for analysis. To analyze the association of IR with primary and secondary outcomes, a univariate logistic regression analysis was conducted, including all variables and comorbidities listed in Table 1, to calculate unadjusted odds ratios (ORs) for the primary outcome. All variables with p-values of less than 0.2 were then included in a multivariate logistic regression model. Table 2 displays the univariate association of variables significant at p<0.2 and subsequent multivariable logistic regression model. A p-value of less than 0.05 was deemed significant in the multivariate analysis. Additional confounders were chosen based on a literature review. The combined Charlson Comorbidity Index (CCI) was utilized to adjust for comorbidity burden. Multivariate logistic and linear regression models were also used to adjust for confounders of the secondary outcomes, with all variables and comorbidities listed in Table 1 included. The odds of the primary outcome was reported as crude OR or adjusted odds ratio (aOR) from the univariate or multivariate analysis, respectively, while the adjusted odds of prolonged hospitalization or increased hospital costs were reported as the incidence risk ratio (IRR).

Ethical considerations

The utilization of the NIS is regulated by the U.S. Agency for Healthcare Research and Quality (AHRQ). To comply with relevant regulations, the NIS design diligently follows the guidelines established in the Healthcare Insurance Portability and Accountability Act (HIPAA) of 1996 and the Omnibus final rule of 2013. Beginning in 2012, the AHRQ took measures to safeguard patient and hospital information by excluding 16 direct identifiers from the NIS dataset, ensuring the highest standards of privacy and security. Consequently, the NIS is categorized as a limited dataset, exempting it from the requirement of Institutional Review Board (IRB) approval.

Data availability statement

All NIS datasets used in the index study are publicly available through the authorized Healthcare Cost and Utilization Project (HCUP) central distributor upon request at https://hcup-us.ahrq.gov/tech_assist/centdist.jsp.

## Results

Sociodemographic characteristics of the study population

The analysis comprised 294,864 hospitalizations for ALD admitted between 2016 and 2018 (Figure 1). Of these hospitalizations, 383 (0.13%) had a secondary diagnosis of IR (incidence rate of 1.3 per 1000 admissions for ALD), while the remaining 294,481 (99.87%) were used as controls. The mean age of patients with ALD and IR was 54 years, compared to ALD without IR (53 years; p<0.001). In addition, the proportion of males was higher in the IR group (74.4%) compared to the controls (67.8%), although this difference was not statistically significant (p=0.234). The majority of patients in both groups were Caucasian (77.6% in the IR group and 67.2% in the control group), followed by Hispanic, African American, and Asian or Pacific Islander races.

Notably, ALD with IR had a higher CCI (84.6% with a score of ≥3) compared to those without IR (73.7% with a score of ≥3). The Midwest region had the highest proportion of ALD with IR (35.9%) compared to the Southern region for ALD without IR (38.3%). Furthermore, the majority of hospitalizations in both groups occurred in teaching hospitals, with 78.2% of patients with IR and 71% of those without IR being admitted to these institutions (Table 1).

**Table 1:** Sociodemographic characteristics of the study population with and without insulin resistance * Table contains a zero in the marginals, statistics cannot be computed. ^a ^All values are significant at <0.05. ALD: alcoholic liver disease; IR: insulin resistance

Patient and hospital variables	ALD without IR	ALD with IR	p-value^a^
Sample size (n = 294,864)	294,481 (99.87)	383 (0.13%)	
Female sex, No (%)	94,822 (32.2)	98 (25.6)	0.234
Mean age, (years)	53	54	<0.001
Race/ethnicity, No (%)			0.219
White	197,891 (67.2)	297 (77.6)	
Black	55,068 (18.7)	15 (4.0)	
Hispanic	51,534 (17.5)	55 (14.5)	
Asian or Pacific Islander	3,828 (1.3)	10 (2.6)	
Native American	6,184(2.1)	0 (0)	
Other	9,570 (3.3)	5 (1.3)	
Charlson comorbidity index score, No (%)			*
0	0 (0)	0 (0)	
1	55,657 (18.9)	49 (12.8)	
2	21,792 (7.4)	10 (2.6)	
≥ 3	217,032 (73.7)	324 (84.6)	
Median annual income in patient’s zip code, US$, No (%)			0.026
1-45,999	95,117 (32.3)	90 (23.4)	
46,000-58,999	72,215 (26.9)	70 (18.2)	
59,000-78,999	68,909 (23.4)	134 (35.1)	
≥ 79,000	51,240 (17.4)	90 (23.4)	
Insurance type, No (%)			0.005
Private including HMO	74,209 (25.2)	152 (39.7)	
Medicare	78,332 (26.6)	118 (30.8)	
Medicaid	110,430 (37.5)	83 (21.8)	
Uninsured	31,509 (10.7)	29 (7.7)	
Hospital region, No (%)			0.007
South	112,786 (38.3)	108 (28.2)	
Midwest	60,663 (20.6)	137 (35.9)	
Northeast	52,712 (17.9)	44 (11.5)	
West	68,614 (23.3)	93 (24.4)	
Hospital bed size, No (%)			0.160
Large	156,958 (53.3)	241 (62.8)	
Medium	85,105 (28.9)	74 (19.2)	
Small	52,417 (17.8)	69 (18)	
Hospital location/teaching status, No (%)			0.377
Rural location	17,669 (6)	15 (3.9)	
Urban location	67,730 (23)	69 (18)	
Teaching hospital	209,081 (71)	300 (78.2)	

Primary outcome

A total of 15,435 mortalities were observed in the study, accounting for 5.23% of the entire cohort. Among them, eight mortalities (0.05%) occurred in the study group and 15,427 (99.9%) in the control group. The unadjusted OR of in-hospital mortality for the study group was 0.98 (95% confidence interval (CI): 0.356-2.686, p=0.965). On the multivariable regression analysis, after adjusting for other confounding factors, there was no significant association between co-existing IR in ALD and the OR of in-hospital mortality (aOR: 1.10, 95% CI: 0.370-3.251, p=0.867) (Table 2).

**Table 2:** Regression analyses of parameters associated with mortality ^a ^Significant at values <0.2. ^b ^Significant at values <0.05. ^c ^Ascites, hepatic encephalopathy, malnutrition, variceal bleeding, hepatorenal syndrome, liver cancer, or need for intensive care. * Only variables with significance level <0.2 on univariate analysis are included in the final multivariate regression. aOR: adjusted odds ratio; OR: crude odds ratio

Variables	Univariate analysis		Multivariate analysis*	
	p-value^a^	OR (95% CI)	p-value^b^	aOR (95% CI)
Insulin resistance	0.965	0.978 (0.356-2.686)	0.867	1.10 (0.370-3.215)
Age	<0.001	1.019 (1.016-1.022)	<0.001	1.018(1.013-1.022)
Female sex	0.966	0.998 (0.924-1.079)	Omitted^*^	
Combined Charlson index	<0.001	1.186 (1.168–1.204)	<0.001	1.128 (1.107-1.150)
High cholesterol levels	0.040	0.818 (0.677–0.987)	<0.001	0.728 (0.634-0.834)
Obesity	0.036	0.750 (0.570-0.981)	0.002	0.801 (0.694-0.921)
Alcohol dependence	0.016	0.681 (0.498-0.932)	0.001	1.144 (1.055-1.240)
Black race	0.180	1.092 (0.960-1.241)	0.991	1.001 (0.873-1.147)
Hispanic	0.015	0.880 (0.794-0.975)	0.045	0.894 (0.802-0.998)
Asian or Pacific islander descent	0.968	0.993 (0.706-1.398)	Omitted^*^	
White American	0.127	1.206 (0.948-1.533)	0.090	1.262 (0.964-1.652)
Lower median household income quartiles	0.614	1.025 (0.932-1.126)	Omitted^*^	
Any complication^c^	<0.001	9.872 (8.626–11.298)	<0.001	9.346 (8.102-10.781)
Weekend admission	0.017	1.106 (1.018-1.203)	0.033	1.104 (1.010-1.209)
Hospitals in the South	0.919	1.006 (0.901-1.123)	Omitted^*^	
Hospitals in Midwest region	0.786	1.017 (0.899-1.151)	Omitted^*^	
Hospitals in the West	0.211	1.163 (1.034-1.308)	Omitted^*^	
Hospital teaching status	<0.001	1.130 (1.056-1.209)	0.182	1.051 (0.977-1.130)
Medium hospital	0.004	1.190 (1.056-1.341)	0.011	1.181 (1.039-1.345)
Large hospitals	<0.001	1.343 (1.204-1.498)	<0.001	1.286 (1.142-1.450)
Uninsured	0.141	0.901 (0.784-1.035)	<0.001	1.408 (1.203-1.647)

However, other factors, such as alcohol dependence, a high CCI score, older age, lack of insurance, incidence of any complication, admission to medium or large hospitals, and weekend admissions, were found to be independently associated with an increased likelihood of mortality (Table 2).

Further analysis revealed that variables, such as sex, lower median household income quartiles for patients’ ZIP code, hospital location, or teaching status, were not significantly associated with the odds of mortality (Table 2).

Secondary outcomes

A total of 60,864 incidences of ALF were recorded, which accounted for 20.64% of the total admissions. The proportion of ALF was found to be similar in both the study and control groups (20.51% vs. 20.64%, respectively). Multivariate logistic regression analysis did not reveal any significant difference between IR in ALD and the likelihood of ALF (aOR: 1.11, 95% CI: 0.371-3.291, p=0.857). However, several independent predictors of ALF were identified on multivariate regression, including older age (aOR: 1.02, 95% CI: 1.013-1.023, p<0.001), a higher CCI (aOR: 1.13, 95% CI: 1.108-1.152, p<0.001), lack of insurance (aOR: 1.41, 95% CI: 1.206-1.660, p<0.001), admission to large hospitals (aOR: 1.28, 95% CI: 1.139-1.450, p<0.001), alcohol dependence (aOR: 1.15, 95% CI: 1.057-1.244, p<0.001), severe sepsis (aOR: 10.92, 95% CI: 9.771-12.214, p<0.001), or the occurrence of any complication during the index hospitalization (aOR: 9.24, 95% CI: 8.001-10.671, p<0.001).

ALD hospitalizations with IR were noted to have longer hospital stays (7.3 days vs. 6.0 days: IRR, 1.17; 95% CI, 1.09-1.26; p<0.001) and higher mean THC ($91,124 vs. $65,290: IRR, 1.32; 95% CI, 1.20-1.46; p<0.001) compared to patients without IR.

Table 3 summarizes the frequency of individual complications by the IR status in the study population. At least 57.3% (168,957) of all hospitalizations for ALD were associated with one or more complications. The frequency of complications was lower in the cohort of ALD with IR compared to controls (55.1% vs. 57.3%). IR was not associated with significant difference in the likelihood of any complication in the study (aOR: 0.83, 95% CI: 0.535-1.274, p<0.001).

**Table 3:** Frequency and odds of complications by the IR status on the multivariate regression analysis ^a ^Significant at values <0.05. ALD: alcoholic liver disease; IR: insulin resistance; aOR: adjusted odds ratio; CI: confidence interval No relationship between iIR and the incidence of ascites, variceal bleeding, or hepatic encephalopathy was found on the statistical analysis.

Complications	ALD without IR, (294,481)	ALD with IR (383)	p-value^a^	aOR (95% CI)
Hepatorenal syndrome	22,027 (7.48)	29 (7.69)	0.944	0.654 (0.256-1.670)
Liver cirrhosis	25,767 (8.75)	29 (7.69)	0.740	0.286 (0.164-1.668)
Liver cancer	8,746 (2.97)	25 (6.41)	0.663	1.306 (0.393-4.346)
Malnutrition	125,507 (42.62)	167 (43.59)	0.858	146 (0.739-1.776)
Critical care admission	35,013 (11.89)	39 (10.26)	0.637	0.836 (0.398-1.758)

## Discussion

IR is a metabolic disorder characterized by the body's reduced ability to respond to insulin, leading to hyperglycemia and impaired glucose metabolism. IR is a well-known risk factor for nonalcoholic fatty liver disease (NAFLD) and has been suggested to play a role in the development of ALD. The extant body of evidence has suggested a potential association between IR and the development of ARLD. However, the index research has diverged from conventional assumptions. The study's findings challenge the prevailing notion that IR, in isolation, significantly exacerbates outcomes for patients hospitalized with ALD. These results prompt a reevaluation of the complex interrelationship between IR and liver diseases, underscoring the need for further investigation into the underlying mechanisms governing this intricate interplay.

It is important to note that the progression of ALD is a multifactorial process, and IR is only one of the many factors that may contribute to disease progression. Other factors, such as oxidative stress, inflammation, and genetic susceptibility, may also play significant roles in the development of ALD [11-13]. Therefore, it is unlikely that IR alone could worsen outcomes for patients with ALD. While IR has been linked to the development of NAFLD [13,14], which shares some similarities with ALD [15], the two diseases differ in several important aspects. For example, NAFLD is often associated with obesity and metabolic syndrome, while ALD is predominantly linked to chronic alcohol abuse. Moreover, NAFLD is typically characterized by the accumulation of fat in the liver, while ALD is characterized by liver inflammation and fibrosis, which may be caused by oxidative stress and immune system dysregulation. Therefore, it is not surprising that the role of IR in the development of NAFLD and ALD may differ significantly [16,17].

Some studies have shown that insulin sensitizers, such as metformin, can improve outcomes in patients with ALD [18-22]. These drugs improve insulin sensitivity and have been shown to reduce liver inflammation and fibrosis, indicating that IR may be a modifiable risk factor for ALD. Few studies have found any significant association between IR and ALD outcomes, suggesting that IR may not be a significant contributor to ALD progression.

Previous research has firmly established a robust correlation between metabolic syndrome and liver diseases, including ALD. The mechanisms underlying this association involve multiple interdependent factors. Metabolic syndrome, characterized by obesity, dyslipidemia, and IR, significantly contributes to the advancement of liver steatosis, inflammation, and fibrosis, which represent key pathological features of ALD [23,24]. While this association indeed holds considerable merit, the findings from the present study suggest that the individual impact of IR as a constituent of metabolic syndrome on clinical outcomes may hold relatively minimal significance. It is more probable that the inadequate management of ensuing diabetes, rather than the mere presence of IR, serves as a more accurate predictor of unfavorable outcomes [25].

The present study is mainly constrained by the nature of the data source. The NIS database, unfortunately, does not provide detailed information about disease severity, which greatly limits our ability to comprehensively assess the impact of ALD or IR severity on the outcomes. In addition, the diagnosis of IR appears infrequently at the time of discharge and is not widely practiced, potentially leading to an underestimation of its prevalence among patients with ALD. Furthermore, the paucity of published studies validating the accuracy of IR diagnosis based on ICD-10 codes adds to the limitations surrounding this assessment. To address these limitations and enhance the reliability of findings, future investigations concerning this crucial question should consider adopting a prospective approach among hospitalized patients, wherein IR can be measured through fasting glucose and insulin levels. Alternatively, a retrospective multicenter chart review of hospitalized ALD subjects, incorporating assessment of IR based on relevant clinical or laboratory variables, could provide valuable insights and allow researchers explore the effects of severity of IR. Despite these limitations, the present study endeavors to attain its research objectives using proven methodologies, offering valuable preliminary insights that could pave the way for future research endeavors in this important area.

## Conclusions

IR is thought to arise from the impact of alcohol on both hepatic and non-hepatic tissues. However, the index study proposes that IR alone might not worsen outcomes for patients with ARLD. It was observed that ALD hospitalizations accompanied by IR led to longer hospital stays and higher hospital costs. The significant statistical association found in this study between IR and increased hospital length of stay, as well as higher mean hospital costs, could be attributed to the burden of comorbidities, the need for additional treatments and monitoring, or other residual confounding variables. Consequently, further investigations are necessary to validate these findings.
